# Decreasing fentanyl concentrations in urinalysis results for methadone treatment admissions: Evidence of changing fentanyl exposure in street drugs

**DOI:** 10.3934/publichealth.2026045

**Published:** 2026-08-07

**Authors:** Emily Giangrande, James A. Swartz

**Affiliations:** Jane Addams College of Social Work, University of Illinois Chicago, Chicago, IL, USA

**Keywords:** fentanyl, opioid overdose, urinalysis, methadone, opioid treatment program, opioids

## Abstract

**Background:**

Recent declines in opioid-related overdose fatalities (OROF) in Chicago and nationally in the U.S. have prompted questions about whether changes in fentanyl exposure may be occurring in the illicit opioid supply.

**Objective:**

This study examined temporal trends in fentanyl exposure among individuals entering opioid treatment, using admission urinalysis as a biological indicator of recent opioid exposure.

**Methods:**

We conducted a secondary analysis of clinical admission data collected between 2023 and 2025 from a community-based opioid treatment program (OTP) on Chicago's West Side (N = 339). Multivariable ordinary least squares regression models were used to examine changes in log-transformed urine concentrations of fentanyl, norfentanyl, and morphine across admission years, adjusting for sociodemographic and clinical characteristics. Concentration analyses were restricted to participants with confirmed positive tests.

**Results:**

Relative to 2023, fentanyl concentrations were significantly lower in 2024 (b = −0.77, p < 0.001) and 2025 (b = −1.29, p < 0.001), corresponding to approximately 54% and 72% reductions, respectively. Norfentanyl concentrations also declined over time, while morphine concentrations remained stable. Participant characteristics and the prevalence of opioid detection at admission were largely stable across years.

**Conclusions:**

Declining concentrations of fentanyl and norfentanyl, alongside stable morphine levels, suggest reduced fentanyl exposure among treatment-seeking individuals over the study period. While urinalysis provides an indirect indicator and cannot establish causality, these findings are consistent with broader reports of declining fentanyl-related overdose mortality and highlight the potential value of treatment-entry toxicology data as a sentinel indicator of changes in illicit opioid markets.

## 1. Introduction

In 2023, the United States experienced the first sustained decline in drug overdose deaths, including opioid-related fatalities, since 2018 [1]–[3]. Final mortality data from CDC WONDER indicate that opioid-related fatalities have steadily declined from 81,250 in 2022 to 79,002 in 2023 and to 53,772 in 2024, corresponding to a reduction in the crude death rate from 24.4 to 15.8 per 100,000 population. Provisional data suggest that opioid-related overdose fatalities (OROF) declined further in 2025, with early partial data indicating 39,931 deaths and a crude rate of 11.7 per 100,000 population [4]. Similar, and in some cases steeper, declines were observed in Illinois and Cook County, where opioid-related mortality has historically been among the highest in the nation [5],[6].

Although there are no definitive explanations for the steep year-over-year declines, several hypotheses have been proposed. One possibility is a reduction in fentanyl presence or concentration in the illicit opioid supply, which has become fentanyl-dominated in many U.S. cities, including Chicago [7]. Recent research suggests that the United States may have experienced a fentanyl supply shock beginning in late 2023 and continuing into 2024, as evidenced by concurrent declines in fentanyl purity, reductions in fentanyl-related drug seizure reports, and increased reports among people who use drugs of fentanyl shortages during this period [8]. The contribution of fentanyl and other non-opioid adulterants (e.g., xylazine, medetomidine) to fatality rates over the past decade has been substantial [9],[10]. The Drug Enforcement Agency (DEA) has attributed recent declines in part to increased enforcement actions targeting drug production and trafficking, which may have altered fentanyl formulation or content in street-level products [3]. In addition, reporting on fentanyl production in Mexico suggests that organizations controlling production have tightened oversight of manufacturing and distribution as a strategy to reduce law-enforcement scrutiny and stabilize prices following saturation [11]. Public health interventions, most notably expanded naloxone distribution, policy changes facilitating buprenorphine prescribing, and shifts in routes of administration, such as reduced injection, may also have contributed to recent reductions in overdose fatalities [12]–[14]. In parallel, federal and state policy changes since 2020 have expanded access to methadone treatment through increased take-home dosing and telehealth-related flexibilities, which may also influence treatment entry patterns and overdose risk.

To examine whether broader changes in the illicit opioid environment are reflected among individuals entering treatment, we analyzed admission urinalysis results from an opioid treatment program (OTP) on Chicago's West Side. Communities in this city area are among the most socioeconomically disadvantaged in the city and are disproportionately composed of racial and ethnic minorities. Historically, they also had the highest rates of illicit opioid use and corresponding OROF [15]. Consistent with national patterns, however, these communities, as well as Chicago and Illinois more broadly, have seen substantial declines in OROF since 2023–2024 [15].

Rather than testing the effects of specific policy interventions or establishing causal pathways to overdose mortality, this study evaluates whether routinely collected treatment-entry toxicology data can function as a sentinel indicator of changes in fentanyl exposure over time. Such data are collected consistently, reflect recent use, and may provide early signals of shifts in illicit drug markets when interpreted alongside other surveillance sources.

We hypothesized that reductions in fentanyl presence or concentration in Chicago's fentanyl-dominated illicit opioid supply would be reflected in admission urinalysis results, with fentanyl detected less frequently and at lower concentrations over time, including norfentanyl, its longer half-life metabolite [16]. If observed changes in the illicit opioid supply were driven primarily by fentanyl rather than broader shifts in opioid use or treatment-seeking populations, we further expected little or no corresponding decline in the detection or concentration of other opioids, such as morphine.

## 2. Materials and methods

This study was a secondary analysis of de-identified clinical data and was determined to be exempt from full review by the University of Illinois Chicago Institutional Review Board. Informed consent was not required, as all data were de-identified prior to analysis.

### 2.1. Participants

Participants were 348 unduplicated adults (age ≥ 18) consecutively admitted to a Chicago-based outpatient methadone treatment program between March 2023 and December 2025. On October 1, 2024, SAMHSA implemented revisions to the standard OTP intake instruments, after which additional admissions were collected using the revised instruments. Variables required for the present analyses were harmonized across instrument versions, and post-October 2024 admissions were appended to the existing dataset prior to analysis. Two admissions with non-binary gender recorded at intake were excluded because gender was included as a covariate in the multivariable regression models, and this category had an insufficient n for stable estimation (remaining N = 346). Seven additional admissions lacked admission urinalysis results and were excluded because the primary outcomes required urinalysis data. The final analytic sample included 339 admissions with complete urinalysis and interview data. Aside from these exclusions, no additional inclusion criteria were applied.

### 2.2. Measures

#### 2.2.1. Opioid concentrations

The primary dependent variables were urine-test concentrations of morphine, fentanyl, and norfentanyl measured at program admission. Admission urinalysis also routinely screened for other opioids, including codeine, non-prescribed methadone (i.e., not dispensed by the OTP at intake and not obtained by prescription from another medical provider), oxycodone, hydrocodone, and norhydrocodone. Because detection of oxycodone, hydrocodone, and norhydrocodone was rare (<3% of admissions), these analytes were excluded from bivariate concentration analyses and multivariable regression analyses. Regression models focused on morphine, fentanyl, and norfentanyl as the most prevalent and policy-relevant opioids. Other opioids were summarized descriptively using detection rates at admission.

#### 2.2.2. Laboratory testing

A third-party laboratory conducted urinalysis using a two-step process. First, specimens were screened using immunoassays that produced binary (yes/no) results indicating the presence of drug metabolites for each drug class (including a fentanyl-class screen). Specimens screening positive underwent confirmatory testing using liquid chromatography–tandem mass spectrometry (LC-MS/MS) to quantify analyte concentrations (ng/mL). Methadone concentrations reflect parent methadone only and do not include concentrations of its primary metabolite, 2-ethylidene-1,5-dimethyl-3,3-diphenylpyrrolidine (EDDP). Both binary detection results and quantified concentrations were used in analyses; concentrations were modeled only among participants with confirmed positive results for each analyte. Buprenorphine was not included in the present analyses because urinalysis results for this analyte were not consistently captured following the October 1, 2024, intake instrument revision; in pre-revision data, buprenorphine detection was rare (<2% of admissions).

#### 2.2.3. Time

Admission year (2023–2025) was the primary predictor in the regression models and was specified as a categorical variable, with 2023 as the reference year.

#### 2.2.4. Sociodemographic and behavioral covariates

Regression models adjusted for sociodemographic and behavioral characteristics collected at admission, including gender (male [reference], female); race/ethnicity (Black/African American [reference], White, Hispanic/Latino, or Other); education (less than high school [reference] vs. high school or higher); employment status (unemployed [reference], employed full- or part-time, or retired/other); Medicaid insurance status (not insured by Medicaid [reference] vs. insured); housing status in the past 30 days (housed [reference] vs. homeless); psychological distress measured using the K-6 and categorized as no or low [reference category], moderate, or severe distress [17]; any overdose in the past 30 days (no [reference], yes); and age in years. Because nearly all (99.2%) participants reported intranasal opiate use, route of administration was not included as a covariate in any analysis.

### 2.3. Procedures

Program treatment staff conducted all admission interviews to obtain self-reported sociodemographic information and obtained urine samples to screen for recent drug use. Urine samples were sent to an external laboratory for the two-step testing process described above, and the results were returned electronically to the clinic. Sociodemographic data and urinalysis results were entered by program staff into a secure, REDCap-hosted electronic database at the University of Illinois Chicago and subsequently downloaded for analysis [18].

### 2.4. Statistical analyses

We conducted all analyses using Stata v.19.5 [19]. Pearson chi-square tests were used to assess bivariate differences in categorical characteristics and opioid detection rates across admission years; OLS regression with admission year as the independent variable was used to test for untransformed concentration values. Concentration analyses were conducted only among participants with confirmed positive urinalysis results for the relevant analyte, as negative screens do not yield meaningful concentration values. Although descriptive analyses (Tables 1 and 2) are based on the full analytic sample (N = 339), sample sizes varied by analyte for concentration analyses (Table 3) and for regression models due to analyte positivity and covariate completeness (Table 4).

Because fentanyl, norfentanyl, and morphine concentrations were right-skewed, these outcomes were log-transformed prior to regression analysis. Regression diagnostics indicated that the log-transformed models satisfied assumptions of normality and homoscedasticity. For concentration models, influential observations were evaluated using Cook's distance (Cook's D > 4/N) on an analyte-specific basis. Exclusion of influential observations did not materially alter coefficient estimates or substantive conclusions; therefore, final regression models retain all eligible observations with confirmed positive tests and complete covariate data for each analyte. Additionally, because 21 participants had urine specimens collected after the admission interview (mean = 10 days post-admission, range = 1–37 days), a sensitivity analysis excluding these cases was conducted to assess the robustness of the findings. Excluding these cases did not materially alter the observed concentrations or the statistical significance of the primary findings; therefore, log-concentration results from the full sample are presented.

Final multivariable OLS regression models included robust standard errors and were adjusted for admission year, gender, race/ethnicity, education, employment status, Medicaid insurance status, housing status, psychological distress, any overdose in the past 30 days, and age (in years). Interaction terms and quadratic age terms were evaluated but were not retained because they did not improve model fit. Participants who tested positive for more than one opioid were retained in all relevant models, as each analyte was analyzed independently. Results are presented as unstandardized regression coefficients with 95% confidence intervals.

## 3. Results

### 3.1. Descriptive analyses

Descriptive characteristics of the analytic sample by admission year are presented in Table 1. Across admission years, participants were primarily male (65.8%), Black/African American (75.4%), had a high school diploma or higher (60.4%), were not experiencing homelessness in the past 30 days (83.3%), were insured by Medicaid (81.4%), and reported moderate psychological distress (54.6%). The mean age at admission was 56.7 years. Most participants had not experienced an overdose in the past 30 days (91.9%). Overall, participant characteristics were largely similar across admission years, although modest differences were observed in the distribution of race/ethnicity and Medicaid insurance status.

### 3.2. Urinalysis detection rates by year

Table 2 presents urinalysis detection rates at admission by year. Fentanyl detection declined across the study period (88.1% in 2023, 92.9% in 2024, and 76.5% in 2025; p < 0.001), and norfentanyl detection showed a similar decline (83.7% in 2023, 89.0% in 2024, and 57.1% in 2025; p < 0.001). In contrast, detection of non-fentanyl opioids, including morphine and codeine, did not differ significantly across admission years.

### 3.3. Detected opioid concentrations among positive tests

Table 3 summarizes the mean urine concentrations detected among participants with confirmed positive opioid analyte tests by admission year. Among fentanyl- and norfentanyl-positive samples, mean concentrations were lower in later admission years, whereas mean morphine concentrations were relatively stable across the study period. As shown, variability in detected concentrations was substantial for all analytes, underscoring the need for multivariable modeling of log-transformed concentrations to assess temporal trends while accounting for individual-level covariates.

### 3.4. Multivariable regression models

Multivariable OLS regression models of log-transformed admission urine concentrations indicated substantial declines in fentanyl and norfentanyl concentrations across admission years, while morphine concentrations remained stable (Table 4). Relative to 2023, mean fentanyl concentrations were approximately 54% lower in 2024 and 72% lower in 2025, whereas norfentanyl concentrations were approximately 32% lower in 2024 and 48% lower in 2025 (percent changes derived by exponentiating coefficients from log-linear models). In contrast, no significant year-to-year differences were observed for morphine concentrations.

These patterns were robust to adjustment for sociodemographic characteristics, psychological distress, Medicaid insurance status, housing instability, and overdose in the past 30 days. Older age was independently associated with lower fentanyl and norfentanyl concentrations, while no covariates showed consistent associations with morphine concentrations. Taken together, these findings indicate that observed temporal declines were specific to fentanyl and its primary metabolite rather than reflecting a generalized change in opioid exposure at admission.

**Table 1.** Sociodemographics by methadone program admission year.

**Admission year**	**2023**	**2024**	**2025**	**Total**	**p-value**
	(N = 86)	(N = 155)	(N = 98)	(N = 339)	
**Gender identity**					
Male	55 (64.0%)	102 (65.8%)	66 (67.3%)	223 (65.8%)	0.889
Female	31 (36.0%)	53 (34.2%)	32 (32.7%)	116 (34.2%)	
**Race-ethnicity**					
Black/African American	71 (82.6%)	120 (77.4%)	60 (65.2%)	251 (75.4%)	0.046
White	3 (3.5%)	10 (6.5%)	11 (12.0%)	24 (7.2%)	
Hispanic	12 (14.0%)	22 (14.2%)	16 (17.4%)	50 (15.0%)	
Other	0 (0.0%)	3 (1.9%)	5 (5.4%)	8 (2.4%)	
**Age in years (mean [SD])**	56.2 [10.0]	57.6 [8.5]	55.8 [10.0]	56.7 [9.3]	0.274
**Education**					
Less than HS	41 (47.7%)	57 (36.8%)	35 (36.8%)	133 (39.6%)	0.206
HS or higher	45 (52.3%)	98 (63.2%)	60 (63.2%)	203 (60.4%)	
**Employment status**					
Unemployed	68 (79.1%)	115 (74.2%)	82 (85.4%)	265 (78.6%)	0.301
Employed full or part-time	8 (9.3%)	20 (12.9%)	8 (8.3%)	36 (10.7%)	
Retired, other	10 (11.6%)	20 (12.9%)	6 (6.2%)	36 (10.7%)	
**Housed or homeless past 30 days**					
No	73 (84.9%)	127 (82.5%)	80 (83.3%)	280 (83.3%)	0.890
Yes	13 (15.1%)	27 (17.5%)	16 (16.7%)	56 (16.7%)	
**Medicaid insurer (y/n)**					
Not insured	16 (18.6%)	21 (13.5%)	26 (26.5%)	63 (18.6%)	0.035
Insured	70 (81.4%)	134 (86.5%)	72 (73.5%)	276 (81.4%)	
**Serious psychological distress**					
No or low	30 (34.9%)	51 (32.9%)	30 (31.2%)	111 (32.9%)	0.937
Moderate	47 (54.7%)	85 (54.8%)	52 (54.2%)	184 (54.6%)	
Severe	9 (10.5%)	19 (12.3%)	14 (14.6%)	42 (12.5%)	
**Any overdose past 30 days**					
No	82 (95.3%)	140 (90.3%)	85 (91.4%)	307 (91.9%)	0.382
Yes	4 (4.7%)	15 (9.7%)	8 (8.6%)	27 (8.1%)	

Note: N = 339 admissions with complete interview and urinalysis data. Sociodemographic data are based on self-reported information collected at admission. Unless otherwise indicated, figures reflect Ns and percents. Percents are based on non-missing data. Significance levels are based on Pearson chi-square tests for categorical variables and on OLS regression for age with admission year as the independent variable and age as the dependent variable.

**Table 2.** Urinalysis detection percentages by opioid analyte at admission year.

**Admission year**	**2023**	**2024**	**2025**	**Total**	**p-value**
	(N = 86)	(N = 155)	(N = 98)	(N = 339)	
**Fentanyl**					
Not present	10 (11.9%)	11 (7.1%)	23 (23.5%)	44 (13.1%)	<0.001
Present	74 (88.1%)	144 (92.9%)	75 (76.5%)	293 (86.9%)	
**Norfentanyl**					
Not present	14 (16.3%)	17 (11.0%)	42 (42.9%)	73 (21.5%)	<0.001
Present	72 (83.7%)	138 (89.0%)	56 (57.1%)	266 (78.5%)	
**Any non-fentanyl opioid** ^†^					
Not present	17 (19.8%)	31 (20.0%)	28 (28.6%)	76 (22.4%)	0.223
Present	69 (80.2%)	124 (80.0%)	70 (71.4%)	263 (77.6%)	
**Morphine**					
Not present	21 (24.4%)	34 (21.9%)	28 (29.2%)	83 (24.6%)	0.433
Present	65 (75.6%)	121 (78.1%)	68 (70.8%)	254 (75.4%)	
**Codeine**					
Not present	54 (62.8%)	89 (57.4%)	65 (66.3%)	208 (61.4%)	0.348
Present	32 (37.2%)	66 (42.6%)	33 (33.7%)	131 (38.6%)	
**Hydrocodone**					
Not present	82 (95.3%)	153 (98.7%)	98 (100.0%)	333 (98.2%)	NE
Present	4 (4.7%)	2 (1.3%)	0 (0.0%)	6 (1.8%)	
**Norhydrocodone**					
Not present	83 (97.6%)	155 (100.0%)	98 (100.0%)	336 (99.4%)	NE
Present	2 (2.4%)	0 (0.0%)	0 (0.0%)	2 (0.6%)	
**Hydromorphone**					
Not present	81 (94.2%)	146 (94.2%)	97 (99.0%)	324 (95.6%)	NE
Present	5 (5.8%)	9 (5.8%)	1 (1.0%)	15 (4.4%)	
**Methadone**					
Not present	37 (43.0%)	81 (52.3%)	38 (39.2%)	156 (46.2%)	0.102
Present	49 (57.0%)	74 (47.7%)	59 (60.8%)	182 (53.8%)	

Note: Urinalysis results are based on testing conducted at treatment admission. Significance levels are based on Pearson Chi-Square tests. Ns for individual tests do not always total 339 owing to missing data for a specific drug. NE = Not estimated when any year has fewer than 10 positive tests for the analyte. ^†^ “Any non-fentanyl opioid” is positive if any of the following were positive: hydrocodone, norhydrocodone, hydromorphone, or morphine.

**Table 3.** Detected concentrations (ng/mL) for positive urinalysis results by opioid at admission year.

**Admission year**	**2023**	**2024**	**2025**	**Total**	**p-value**
	Mean [SD]	Mean [SD]	Mean [SD]	Mean [SD]	
**Fentanyl**	1735.1 [2078.3]	872.9 [1069.6]	565.2 [802.1]	1010.3 [1411.3]	<0.001
**Norfentanyl**	7048.1 [8244.8]	4626.3 [4881.4]	4027.7 [4396.2]	5155.8 [5994.2]	<0.001
**Morphine**	3494.0 [4932.4]	3714.8 [6263.8]	3955.6 [7318.6]	3724.6 [6252.3]	0.913
**Codeine**	394.9 [454.3]	354.8 [447.4]	545.2 [1023.5]	412.6 [642.9]	0.378
**Hydromorphone**	100.4 [44.5]	157.4 [250.8]	60.7 [NE]	132.0 [194.1]	0.832
**Methadone**	2547.6 [3287.8]	2647.8 [3126.7]	2955.3 [3729.2]	2724.6 [3367.9]	0.803

Note: All results reflect ng/mL and show mean and standard deviation values. The Ns for each drug are based on the positive test results for that drug. By drug, the total ns across years were fentanyl (N = 293), norfentanyl (N = 266), morphine (N = 254), codeine (N = 131), hydromorphone (N = 15), and methadone (N = 182). The following drugs were excluded owing to either a small total N (<10) or no positive tests in one or more admission years: hydrocodone, norhydrocodone, and oxycodone. NE = standard deviation not estimable due to n = 1 positive test in 2025.

**Table 4.** OLS regression models of log-transformed admission urine concentrations.

**Analyte**	**Morphine** (N = 251)	**Fentanyl** (N = 273)	**Norfentanyl** (N = 263)
**Admission year (2023 = reference)**			
2024	−0.147	−0.771***	−0.385
	[−0.735, 0.442]	[−1.158, −0.385]	[−0.780, 0.010]
2025	−0.143	−1.289***	−0.645***
	[−0.725, 0.439]	[−1.744, −0.835]	[−1.127, −0.162]
**Gender identity (male = reference)**			
Female	−0.013	0.078	−0.011
	[−0.466, 0.441]	[−0.256, 0.412]	[−0.379, 0.357]
**Race-ethnicity (Black/African American = reference)**		
White	0.169	−1.052**	−0.599**
	[−0.899, 1.237]	[−1.686, −0.419]	[−1.032, −0.167]
Latino, Hispanic	−0.186	−0.644*	−0.499
	[−0.830, 0.458]	[−1.184, −0.104]	[−1.048, 0.050]
Other	−0.204	−0.460	0.337
	[−1.486, 1.077]	[−1.388, 0.468]	[−0.346, 1.019]
**Education (less than HS = reference)**	
HS or higher	−0.064	0.255	0.068
	[−0.473, 0.345]	[−0.087, 0.596]	[−0.290, 0.425]
**Housed or homeless past 30 days (housed = reference)**		
Homeless	−0.236	−0.153	0.020
	[−0.870, 0.398]	[−0.658, 0.352]	[−0.406, 0.446]
**Employment status (unemployed = reference)**		
Employed full or part-time	0.189	0.183	0.263
	[−0.456, 0.835]	[−0.352, 0.718]	[−0.262, 0.789]
Retired, other	0.231	0.227	0.084
	[−0.430, 0.892]	[−0.397, 0.851]	[−0.435, 0.603]
**Insured by Medicaid (not insured = reference)**		
Insured	0.692*	−0.019	−0.099
	[0.137, 1.247]	[−0.557, 0.519]	[−0.578, 0.381]
**Any overdose past 30 days**			
Yes	−0.560	−0.034	−0.287
	[−1.195, 0.075]	[−0.788, 0.720]	[−0.745, 0.170]
**Serious psychological distress (no or low = reference)**		
Moderate	0.107	−0.148	−0.185
	[−0.329, 0.542]	[−0.513, 0.218]	[−0.584, 0.213]

Severe	0.212	−0.005	−0.243
	[−0.558, 0.981]	[−0.552, 0.542]	[−0.880, 0.394]
**Age in years (centered)**	0.002	−0.050***	−0.037**
	[−0.027, 0.031]	[−0.072, −0.028]	[−0.059, −0.015]
**R^2^**	0.051	0.203	0.093

Note: All results are based on OLS regressions estimated with robust standard errors. Results shown are the unstandardized regression weights and 95% confidence intervals shown in brackets. Ns for each drug reflect those with a positive test for that drug and exclude those not tested or who tested negative or were missing one or more of the included covariates. ***p < 0.001, **p < 0.01, *p < 0.05.

## 4. Discussion

This study examined changes in urinalysis concentrations of fentanyl, norfentanyl, and morphine among adults entering an opioid treatment program on Chicago's West Side between 2023 and 2025. We observed substantial and consistent year-over-year declines in both fentanyl and norfentanyl concentrations, while morphine concentrations remained stable across admission years. This pattern indicates that changes in opioid exposure at treatment entry were specific to fentanyl rather than reflecting a generalized shift across opioids. Although the toxicology data cannot distinguish heroin-derived morphine from pharmaceutical morphine use, the study population and prior research conducted among opioid-using populations in Chicago suggest that many morphine-positive specimens likely reflect heroin use or heroin-fentanyl mixtures rather than prescribed morphine products [20]. The observed reductions in fentanyl and norfentanyl concentrations among study participants occurred during a period of declining opioid-related overdose fatalities in Chicago and nationally [1]–[5],[13], highlighting the potential relevance of treatment-entry toxicology data for contextualizing broader overdose trends. While these findings cannot be directly linked to a decrease in fentanyl availability in the illicit drug supply, they support recent work suggesting a supply shock that reduced fentanyl supply in the U.S. [8].

Our findings are based on urinalysis results collected at treatment entry and therefore do not distinguish intentional from unintentional fentanyl exposure, nor do they capture timing or quantity of recent use. Although patient-reported opioid of choice was not available in the present dataset, prior studies conducted among opioid-using populations recruited from the same Chicago communities and syringe service programs provide useful context. In one study, nearly all participants identified heroin as their preferred opioid despite widespread fentanyl exposure confirmed by urinalysis [21]. Similarly, a community-based drug-checking study found that fentanyl exposure was nearly ubiquitous, while preference for fentanyl was substantially less common [20]. Taken together, these findings suggest that fentanyl exposure in this population frequently occurs through a drug supply in which fentanyl is commonly present in products sold as heroin or heroin-fentanyl mixtures rather than through intentional fentanyl use alone. Nevertheless, the concurrent decline in the prevalence of fentanyl detection and the more pronounced decline in fentanyl concentrations over time remain consistent with reduced fentanyl exposure among treatment-seeking individuals during the study period.

The reasons for the observed reductions in fentanyl exposure remain debated and are likely multifactorial. Proposed contributors include restrictions on precursor chemicals, intensified U.S. and Mexican law-enforcement actions targeting fentanyl production and trafficking, and reported shifts in cartel manufacturing practices toward pill-based products. In parallel, drug seizure data indicate increasing prevalence of non-opioid adulterants such as xylazine and medetomidine, which may reflect market adaptations occurring alongside changes in fentanyl availability [22]. Taken together, these contextual factors highlight the complexity of attributing causality while outlining plausible mechanisms that are consistent with—but not determinative of—the temporal patterns observed in this study.

Alternative explanations for recent declines in opioid-related overdose fatalities have emphasized mechanisms that reduce mortality risk without necessarily altering fentanyl exposure itself. These include harm-reduction interventions such as expanded naloxone distribution, which can lower the likelihood of fatal overdose without changing patterns of fentanyl use or potency. Other proposed explanations include increased tolerance among a more experienced population of opioid users and changes in the size or composition of the opioid-using population following prolonged periods of high overdose mortality. While such factors may plausibly contribute to observed declines in fatal overdoses, they do not directly address the concurrent reductions in fentanyl detection and concentration observed at treatment entry in this study [23].

The observed decreases in fentanyl concentrations and contemporaneous declines in opioid-related overdose fatalities are encouraging following years of sustained increases. However, the durability of these trends remains uncertain. Historical patterns suggest that apparent reductions in illicit opioid supply can be temporary and vulnerable to rapid reversal. Recent analyses have proposed that observed changes in fentanyl availability may reflect shifts in production practices and supply-chain organization rather than a sustained contraction of the illicit opioid market. In particular, greater consolidation of fentanyl production by transnational organizations has been hypothesized as a mechanism that could temporarily constrain supply while enabling longer-term stabilization or re-expansion under more centralized control. Although such hypotheses are difficult to test empirically, they underscore the importance of ongoing surveillance and caution against assuming that recent reductions in fentanyl exposure will necessarily persist.

Finally, although this study cannot establish causality, several features of the data support the interpretation that observed trends reflect changes specific to fentanyl exposure rather than broader shifts in sample composition or measurement. Client demographics, intake procedures, and laboratory methods remained stable across admission years, reducing the likelihood that methodological changes explain the findings. In addition, morphine concentrations remained stable while fentanyl and norfentanyl concentrations declined substantially, providing an internal comparison that strengthens the inference that observed changes were fentanyl-specific.

### 4.1. Limitations

This study has several limitations. First, the analysis is observational and does not establish causal relationships between changes in fentanyl exposure and broader overdose trends. Data were drawn from admissions to a single opioid treatment program on Chicago's West Side and may not represent individuals who use opioids but do not enter treatment; accordingly, findings may not generalize to other populations or geographic settings.

Second, urinalysis provides an indirect indicator of recent drug exposure and does not capture timing, dose, or intent of use. Concentrations are influenced by factors such as the interval between last use and specimen collection, individual metabolic variation, and patterns of polysubstance use. In addition, toxicology data were collected at a single point in time at treatment entry and cannot characterize longer-term exposure trajectories.

The laboratory testing panel did not include emerging adulterants such as xylazine or medetomidine; therefore, temporal changes in these substances could not be evaluated. Recent surveillance reports from the Centers for Disease Control and Prevention and the Chicago Department of Public Health have documented the emergence of medetomidine and other novel adulterants in Chicago's illicit drug supply [24],[25]. Future studies incorporating broader toxicological testing panels may help clarify whether changes in fentanyl exposure occur alongside shifts in other substances present in the illicit opioid supply.

Finally, while the temporal patterns observed here occur alongside broader changes documented in national surveillance and seizure data [3],[6],[26], these external sources are not directly linked to the present analysis and should be interpreted as contextual rather than confirmatory.

## 5. Conclusions

In this study, fentanyl detection rates declined modestly across admission years, whereas fentanyl and norfentanyl concentrations declined substantially. This pattern indicates that while fentanyl remained present among individuals entering treatment, the intensity of fentanyl exposure at admission decreased over time. The stability of morphine concentrations alongside declining fentanyl concentrations suggests that observed changes were fentanyl-specific rather than reflecting generalized substitution or dilution across opioids.

These temporal patterns occurred during a period of declining fentanyl-related overdose fatalities reported at the county and national levels. Although urinalysis data provide only an indirect indicator of drug exposure and cannot establish causal pathways, the findings highlight the potential utility of routinely collected treatment-entry toxicology data as a sentinel indicator of changes in fentanyl (and other drug) exposure. Replication of similar analyses across additional treatment settings and jurisdictions will be important for assessing generalizability, clarifying mechanisms, and informing ongoing surveillance and public health responses.

## Use of AI tools declaration

The authors declare they have not used Artificial Intelligence (AI) tools in the creation of this article.
